# The effect of high-permittivity pads on specific absorption rate in radiofrequency-shimmed dual-transmit cardiovascular magnetic resonance at 3T

**DOI:** 10.1186/s12968-015-0188-z

**Published:** 2015-09-19

**Authors:** Wyger M. Brink, Johan S. van den Brink, Andrew G. Webb

**Affiliations:** C.J. Gorter Center for High Field MRI, Department of Radiology, Leiden University Medical Center, Leiden, The Netherlands; Philips Healthcare, Best, The Netherlands

**Keywords:** High permittivity materials, Specific absorption rate, Cardiovascular magnetic resonance, Dual-channel transmit, RF homogeneity

## Abstract

**Background:**

Dual-channel transmit technology improves the image quality in cardiovascular magnetic resonance (CMR) at 3 T by reducing the degree of radiofrequency (RF) shading over the heart by using RF shimming. Further improvements in image quality have been shown on a dual-transmit system using high permittivity pads. The aim of this study is to investigate the transmit field (*B*_1_^+^) homogeneity and the specific absorption rate (SAR) using high permittivity pads as a function of the complete range of possible RF-shim settings in order to gauge the efficacy and safety of this approach.

**Methods:**

Electromagnetic (EM) simulations were performed in five different body models using a dual-transmit RF coil, with and without high permittivity pads. The RF shimming behaviour in terms of *B*_1_^+^ homogeneity and local SAR were determined as a function of different RF-shim settings. Comparative experimental data were obtained in healthy volunteers (*n* = 33) on either a standard-bore (60 cm diameter) or wide-bore (70 cm diameter) 3 T CMR system.

**Results:**

EM simulations and experimental data showed higher (*B*_1_^+^) homogeneity and lower SAR for optimized RF-shim settings when using the high permittivity pads. The power distribution between the two channels was also much closer to being equal using the pads. EM simulations showed that for all five body models studied, optimized RF-shim settings corresponded to reduced local SAR using high permittivity pads. However, there are also specific, non-optimal RF-shim settings for which the actual SAR using the pads would be higher (up to ~20 %) than that calculated by the CMR system.

**Conclusions:**

The combination of active (dual transmit) and passive (high permittivity pads) RF shimming shows great promise for increasing image quality for cardiac imaging at 3 T. Optimized RF-shim settings result in increased *B*_1_^+^ homogeneity and reduced SAR with the high permittivity pads: however, there are non-optimal cases in which SAR might be underestimated, and these merit further investigation.

## Background

A number of advantages of performing cardiovascular magnetic resonance (CMR) at 3T compared to 1.5T have been reported [1], particularly in applications such as myocardial tagging and perfusion [2, 3] as well as whole-heart coronary artery imaging [4]. However, significant challenges remain in performing routine clinical imaging at 3T, especially the reduced radiofrequency (RF) transmit field (*B*_1_^+^) homogeneity over the heart at 3T compared to 1.5T: Sung et al. [5] have reported that the magnitude of the *B*_1_^+^ field can vary by up to 50 % at 3T. The *B*_1_^+^ inhomogeneity can produce significant shading artifacts leading to a reduced signal-to-noise ratio (SNR) and contrast over the heart [6]. Other issues at 3T include the increased specific absorption ratio (SAR), which limits the minimum repetition time (TR) that can be used, and increased static field (*B*_0_) inhomogeneity over the heart which, in combination with the higher TR, can introduce significant banding into images acquired with the highly efficient steady-state free precession (SSFP) sequences which are used at 1.5T [7].

Recently, all major CMR manufacturers have introduced dual-channel transmit systems for 3T [8]. Although a single-channel system driven in quadrature, i.e. the two modes of the body coil being driven by equal amplitudes and a fixed 90° phase difference, produces a sufficiently homogeneous *B*_1_^+^ field at field strengths up to and including 1.5T, this approach is not optimal at 3T as the reduced RF wavelength, which is approximately 25 cm in tissue at 3T, leads to significant *B*_1_^+^ nonuniformities within the human body. An elliptical drive, i.e. using equal amplitudes but a fixed non-90° phase difference between the two ports, improves this situation in abdominal imaging but does not provide optimal settings for cardiac imaging. The dual-channel transmit approach utilizes two dedicated transmit chains, which allows independent control of the amplitudes and phases driving the two ports of the body coil. The process of optimizing image quality by adjusting the relative amplitudes and phases of these two channels is referred to as “RF-shimming”. A short calibration scan (typically lasting less than a minute) is performed at the beginning of each examination to map the *B*_1_^+^ fields of each of the two channels, and based upon these data the optimal relative amplitudes and phases are calculated. SAR estimates are then obtained from a precalculated SAR database, which includes multiple body models and RF-shim settings. This procedure allows for subject-specific driving conditions that optimize the *B*_1_^+^ homogeneity as well as reduce the SAR [9, 10]. A number of studies have shown that the dual-channel approach increases the *B*_1_^+^ homogeneity over the heart, improves the contrast-to-noise ratio (CNR) between the basal inter-ventricular septum and blood pool, and also between papillary muscles and/or myocardium and the blood pool, and reduces the signal drop-off in the septum, right ventricle and right ventricular free wall [11–13]. In addition, due to the reduced SAR, the TR can be significantly reduced, which leads to a decrease in banding artifacts from SSFP sequences. In one particular study on high-dose dobutamine stress (HDDS) at 3T, Strach et al. [14] found significant improvements in image and diagnostic quality using a dual-channel transmit system compared to operating in single-channel mode. In their study 13/13 cases were found to be diagnostic in dual-channel transmit mode, compared to only 5/13 in single-channel transmit mode.

An alternative (but also complementary) approach to improving image quality is the use of high permittivity materials [15]. The underlying physical mechanism is that these materials support a strong and localized density of electrical displacement currents that are induced by the primary RF field from the body coil. These currents can be thought of as secondary sources which superimpose a secondary RF field onto the primary RF field present without high permittivity pads. Therefore, introducing a high permittivity material will generally lead to a local enhancement of the *B*_1_^+^ field, which can be tailored to a specific organ or region-of-interest. Previous results have shown that two high permittivity pads optimized for cardiac imaging can significantly increase the CNR, reduce SAR by as much as 50 % and improve *B*_1_^+^ homogeneity in functional cardiac magnetic resonance at 3T [16]. However, the latter two characteristics have not explicitly been studied as a function of the RF-shim settings, i.e. the relative phases and amplitudes used during the scan. Since the SAR model used in commercial CMR systems obviously does not account for the presence of high permittivity pads, it is currently unknown if the presence of pads while scanning can lead to actual SAR levels higher than those reported by the system under certain conditions of RF-shimming. The aim of this study is to perform a thorough analysis of the *B*_1_^+^ and SAR performance of a 3T dual-channel transmit system as a function of RF-shimming in order to address these questions.

## Methods

Healthy volunteers were scanned under a protocol approved by the local institutional review board. Signed informed consent was obtained from all volunteers.

### Electromagnetic simulations

The transmit RF field in a two-port high pass birdcage body coil tuned to 128 MHz was simulated using commercially available software (xFDTD, version 7.2, Remcom inc., State College, PA, USA). The dimensions of the coil were 50 × 61 cm (length × diameter), and the shield had a diameter of 67 cm. These dimensions correspond to the body coil on a standard-bore (60 cm diameter) 3T system. An isotropic grid of 2.5 mm^3^ was used with mesh refinement up to 1 mm around the dielectric pads. A set of five body models were simulated with a body-mass-index (BMI) ranging from 19.2 to 27.3 kg/m^2^. Two body models were obtained from the Virtual Family dataset [17], “Duke” (BMI = 23.1 kg/m^2^) and “Ella” (BMI = 22.0 kg/m^2^) and three body models from the GSF Family [18], “Golem” (BMI = 22.2 kg/m^2^), “Irene” (BMI = 19.2 kg/m^2^) and “Donna” (BMI = 27.3 kg/m^2^).

The simulated field data were processed to evaluate the RF shimming behaviour in terms of *B*_1_^+^ inhomogeneity over the whole heart (expressed as the coefficient-of-variation CV_*B*1_^+^, the ratio of the standard deviation to mean value), and local torso SAR, defined as the peak 10 g-averaged SAR within the torso. These measures were calculated for a range of absolute phase differences (−90° to +270°) and relative power ratios (−20 dB to +20 dB; i.e. amplitude ratios of 0.1 to 10) of the two channels. A Q-matrix formalism was applied to model local SAR during RF shimming [19], and 10-g averaging of the Q-matrices was performed using a FFT-based kernel growing method [20]. All routines were implemented in Matlab (version 2012, Mathworks, Natick, MA). All field data were normalized to the average *B*_1_^+^ in the transverse cross-section of the body (i.e. a global rather than local *B*_1_^+^ value), which corresponds to the *B*_1_^+^ calibration procedure that is performed on the scanner [21].

### High permittivity pads

Two high permittivity pads, with a relative permittivity of ~300 and conductivity of ~0.4 S/m, were formed as described previously [16] using a suspension of barium titanate (−325 mesh powder, Alfa Aesar, The Netherlands) in distilled deionized water. The dimensions of the high permittivity pads were 20-by-20 cm, with a thickness of 1.5 and 1.0 cm for the anterior and posterior pad, respectively. The pads were positioned centered at the heart. After fast localizer scans were acquired in both the transverse as well as sagittal planes in order to visualize the position of the pads in the x- and z-directionswith respect to the heart, a manual adjustment of their position was performed if their position was off by more than ~3 cm.

### CMR protocol

Experimental data were acquired on two different systems, a “standard bore” 60 cm diameter and a “wide bore” 70 cm diameter, to provide a comprehensive assessment of the performance in a cohort of 11 and 22 healthy volunteers, respectively (the cohort of 11 volunteers on the 60 cm diameter bore are the same as those as in [16]. The imaging protocol, comprising scout images, RF-shimming on the heart, and cine-acquisitions in short- and long-axis with and without high permittivity pads were also described in [16]. The RF-shim settings were recorded for each volunteer both in the short-axis and four-chamber views, meaning that two different settings were collected for each subject. The BMI values for the subjects ranged from 20.1 to 34.9 kg/m^2^, and body surface area (BSA) from 1.56 to 2.44 m^2^.

## Results

**Fig. 1:**
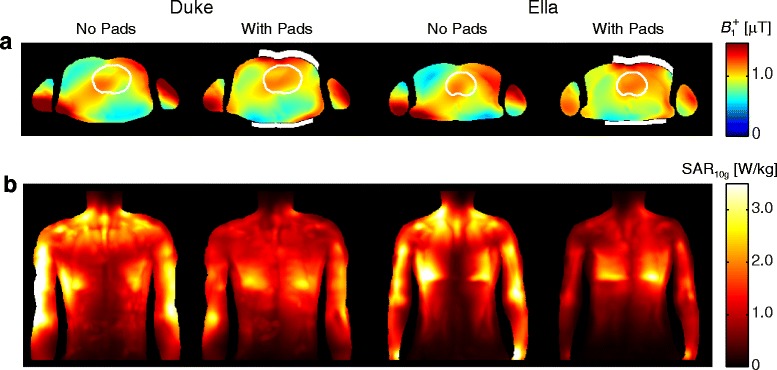
Simulations of the RF-shimmed dual-channel transmit system (standard bore) in the body models “Duke” (*left*), and “Ella” (*right*) with and without high permittivity pads, shown in white. **a** Transverse cross-sections of the *B*_1_^+^ and **b** coronal maximum intensity projections of the 10 g-averaged SAR. The heart is outlined by the thin line. The phase settings are those calculated for maximum *B*_1_^+^ homogeneity for each individual case (see Fig. 2). The pads improve the *B*_1_^+^ homogeneity within the heart and significantly reduce local SAR throughout the body. All data are normalized to a mean *B*_1_^+^ of 1 μT in the transverse cross-section of the body model

Figure 1 shows simulated *B*_1_^+^ and SAR maps two selected body models in the standard-bore magnet, using the RF-shim settings which optimize the *B*_1_^+^ homogeneity within the heart for each case. Results obtained in a model of the body coil used in the wide-bore system essentially show very similar results (data not shown). This figure illustrates the improved *B*_1_^+^ homogeneity and the reduced SAR that can be achieved when using the high permittivity pads in combination with optimized RF-shim settings.

**Fig. 2:**
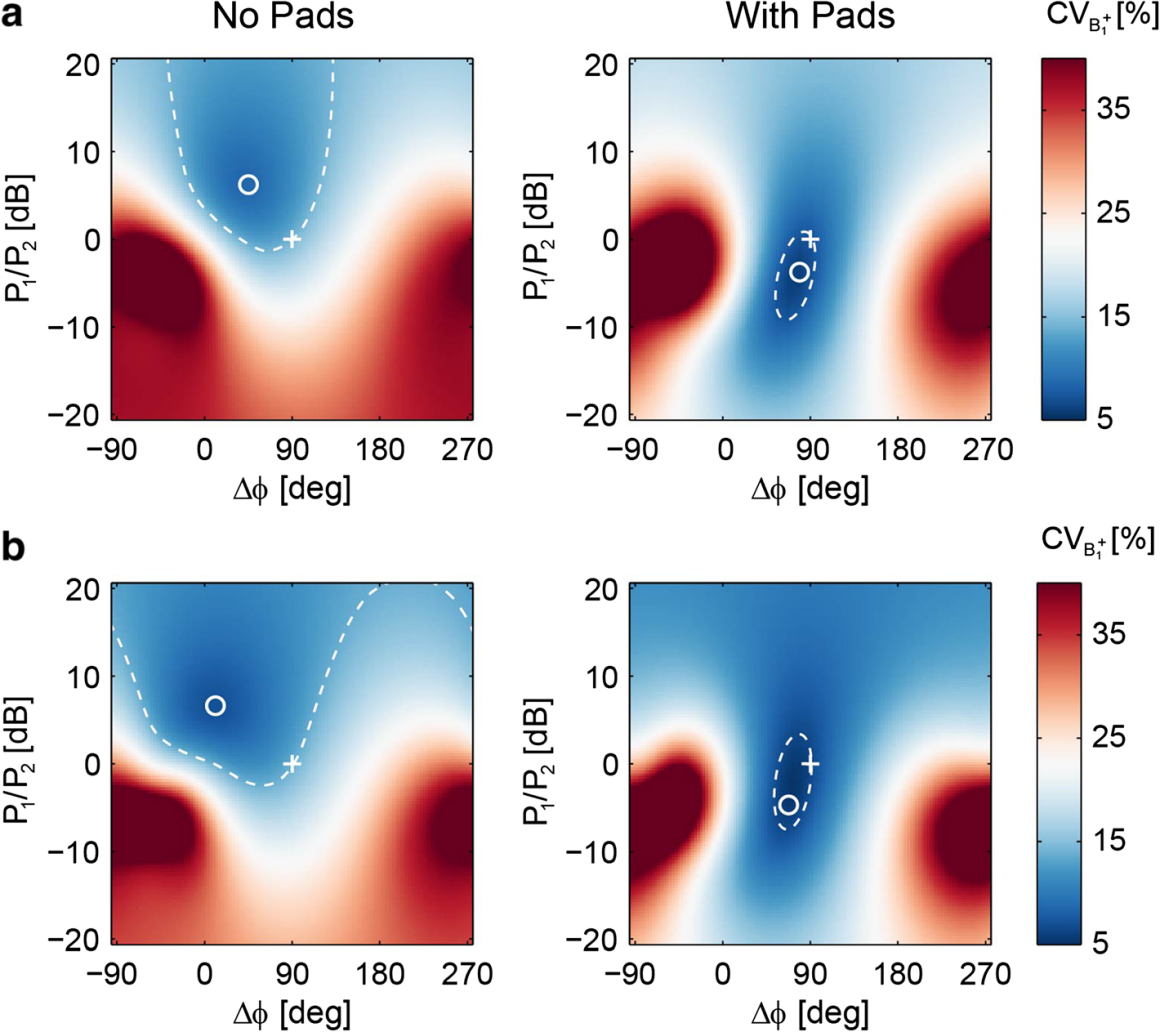
Two-dimensional plots of the *B*_1_^+^ inhomogeneity (CV_*B*1+_) with RF-shim settings simulated in the body models “Duke” (**a**), and “Ella” (**b**) with and without high permittivity pads. In each plot, quadrature mode is indicated by the plus sign (+) and the RF-shim setting that maximizes *B*_1_^+^ homogeneity is indicated by the circle (*o*). The dashed line indicates the iso-contour at the level of *B*_1_^+^ homogeneity obtained in quadrature mode

Figure 2 shows the effect of different RF-shim settings, i.e. the relative power ratio and phase difference of the two channels, on the inhomogeneity of the *B*_1_^+^ field (measured as CV_*B*1+_) over the heart. Figure 2a shows that, for the Duke model without pads, the highest *B*_1_^+^ homogeneity (white circle) occurs at a phase difference of approximately 45° and a power ratio of approximately 8 dB (i.e. a signal amplitude ratio of 2.5 between the two channels). For these settings the CV_*B*1+_ is 8.6 % compared to 14.4 % for when the coil is driven in quadrature (shown as a white plus sign). The isocontour, drawn at the level of *B*_1_^+^ homogeneity obtained in quadrature mode, indicates that a large range of RF-shim settings result in an improved transmit homogeneity compared to quadrature mode. The behaviour with the high permittivity pads in place is quite different. With the RF-shim settings optimized for the highest *B*_1_^+^ homogeneity corresponding to a power ratio close to unity, and a phase difference ~90°; i.e. the RF coil being driven in quadrature mode. With these settings, the CV_*B*1+_ is 5.9 %, which reflects a further improvement of the *B*_1_^+^ homogeneity due to the pads. The isocontour line in this case indicates a very tight set of well-defined RF-shim settings (compared to the much more “diffuse” case without pads) would improve the *B*_1_^+^ homogeneity over the quadrature driven body coil. The qualitative behavior is very similar for the other body models, as also shown for the Ella model in Fig. 2b, with the relevant numbers reported in Table 1.

**Table 1:** Overview of *B*_1_^+^ inhomogeneity (CV_*B*_1+) for different RF-shim settings

RF-Shim	Duke		Ella		Golem		Irene		Donna	
	No Pads	With Pads	No Pads	With Pads	No Pads	With Pads	No Pads	With Pads	No Pads	With Pads
Quadrature	14.4 %	7.4 %	13.8 %	6.4 %	10.7 %	8.5 %	11.5 %	8.1 %	15.0 %	8.0 %
Minimum CV_B1+_	8.6 %	5.9 %	7.1 %	5.4 %	8.2 %	7.9 %	6.8 %	7.3 %	7.5 %	6.4 %
Minimum local SAR	14.2 %	7.9 %	11.5 %	7.8 %	11.0 %	9.1 %	10.1 %	9.6 %	11.9 %	6.4 %

**Fig. 3:**
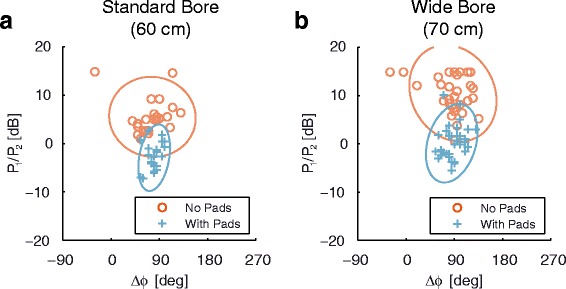
Experimental RF shimming results acquired on the standard bore (**a**) and wide bore (**b**) system, together with corresponding 95 % confidence ellipses. RF-shim settings for images acquired in the long- and short-axis orientations were recorded separately

Experimental measurements of the RF-shim settings determined by the system to optimize the *B*_1_^+^ homogeneity over the heart are shown in Fig. 3, which plots the relative power ratios and phase differences measured on both the standard-bore and wide-bore systems. Confidence ellipses are shown at a level of 95 %. Although there is significant variation between individual subjects, a large imbalance in the power delivered to the two channels is clear when no pads are used, whereas the optimal RF-shim when the high permittivity pads are used is close to the quadrature case.

**Fig. 4:**
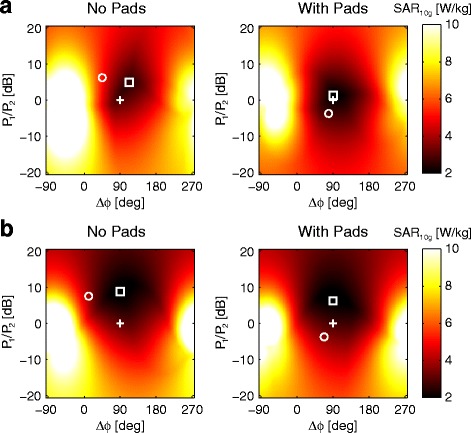
Variation of local torso SAR with RF-shim settings simulated in the “Duke” (**a**) and “Ella” (**b**) body models, with and without high permittivity pads. In each plot, quadrature mode is indicated with the plus sign (+) and the shim setting which minimizes local torso SAR by the box (*□*). For reference, the shim setting that maximizes *B*_1_^+^ homogeneity is indicated by the circle (*o*), as previously shown in Fig. 2. All data are normalized to the mean *B*_1_^+^ in the transverse cross-section of the body

Figure 4 shows the variation of local torso SAR in two selected body models as a function of RF-shim setting. In the case of the Duke model without the pads, the lowest SAR value of 2.5 W/kg is found at about a 5 dB power ratio and a 110° phase difference. However, when using these RF-shim settings the CV_*B*1+_ is 14.2 %, essentially the same as in the quadrature case. When the pads are added, the lowest SAR value is reduced to 1.9 W/kg, and the corresponding CV_*B*1+_ using these RF-shim settings is 7.9 %. These values, and those obtained using the other body models are shown in Tables 1 and 2. A summary of the corresponding RF-shim settings is reported in Table 3.

**Table 2:** Overview of local torso SAR in W/kg for different RF-shim settings

RF-Shim	Duke		Ella		Golem		Irene		Donna	
	No Pads	With Pads	No Pads	With Pads	No Pads	With Pads	No Pads	With Pads	No Pads	With Pads
Quadrature	3.0	2.1	3.2	2.8	2.8	2.5	3.3	2.9	2.9	2.4
Minimum CV_B1+_	5.3	2.8	4.1	3.4	3.9	3.0	4.1	3.5	7.0	1.8
Minimum local SAR	2.5	1.9	1.8	1.8	2.0	1.9	2.0	2.0	1.9	1.7

**Table 3:** Summary of simulated RF-shim settings

RF-Shim	No Pads		With Pads	
	P_1_/P_2_ [dB]	Δϕ [deg]	P_1_/P_2_ [dB]	Δϕ [deg]
Minimum CV_*B*1+_	7.3 ± 2.8	18.7 ± 27.9	−1.8 ± 3.9	74.3 ± 6.2
Minimum local SAR	6.9 ± 1.8	101.3 ± 8.9	4.5 ± 2.3	90.0 ± 6.0

Values are means ± standard deviations (*n* = 5)

**Fig. 5:**
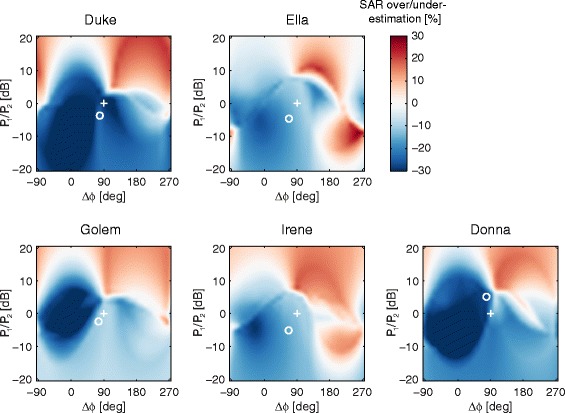
Plots of the over- or underestimation of SAR when using high permittivity pads. Blue refers to overestimation, i.e. the actual SAR is less than that calculated by the CMR system. Quadrature mode is indicated with the plus sign (+) and the RF-shim setting that maximizes *B*_1_^+^ homogeneity is indicated by the circle (o)

The results in Fig. 5 address the question of to what extent high permittivity pads change the SAR behavior during RF shimming, given that the SAR values reported on the scanner are calculated based on models that do not incorporate high permittivity pads. For example, from Tables 1 and 2, if the system operates in quadrature mode with pads present, the SAR for the Duke model without pads would be 3.0 W/kg, whereas the actual SAR is 2.1 W/kg. So, the reported value would be an overestimation of ~30 % and there would be no safety concerns operating in this mode: similarly for the other models. Then, if in the case of the Duke model RF shimming were performed to maximize the *B*_1_^+^ homogeneity, then the reported SAR value would be 3.8 W/kg, whereas the actual value is 2.8 W/kg; which reflects again an overestimation of ~25 %. However, there are also RF-shim settings for which the reported value is less than the actual value, although these correspond to RF-shim settings which are sub-optimal in terms of either *B*_1_^+^ homogeneity or SAR. Figure 5 shows the degree to which SAR is over- or underestimated as a function of RF settings for all five different body models, with a wide range of BMI values, heights and weights. In all cases quadrature operation results in a conservative SAR estimate when based on the situation without pads. In four of the five cases, the RF-shim settings which correspond to the highest *B*_1_^+^ homogeneity correspond to points very far removed from the areas in which SAR would be underestimated (shown in red). In the case of Donna, which has the highest BMI of 27.3 kg/m^2^, optimal RF shimming still results in a lowered SAR compared to that reported, but there is quite a sharp transition to a set of RF shims which would underestimate the SAR.

## Discussion

The results of this study show that, when combining high permittivity pads and dual-channel RF shimming, then in general not only is the *B*_1_^+^ uniformity over the heart increased, but also the local SAR is reduced. An important observation of the simulated and experimental data is that the optimal RF-shim settings, in terms of minimizing *B*_1_^+^ inhomogeneity or SAR, are much closer to quadrature settings when high permittivity pads are used. This has several advantages: one is the much more equal amounts of power required by both channels, meaning that the upper power limit of the RF amplifier is not reached as quickly as in the case of the much greater degree of power imbalance when high permittivity pads are not used. A second is that the process of optimization of the RF-shim values is a much more well-defined problem than without the pads: as shown in Fig. 2 the optimum value with pads lies very close to the starting point (which is typically quadrature) and the region of the CV_*B*1+_ minimum is much more tightly defined than in the case without pads. The mechanism behind this is that the high permittivity pads can be thought of as making the elliptical subject more circulo-symmetric, which means that each channel of the body coil is equally efficient.

As shown in Fig. 5, for five different body models, the optimization of RF-shim settings either to obtain the lowest CV_*B*1+_ or the lowest SAR, in each case results in an actual SAR with the pads present being lower than that used by the scanner to check the safety of the imaging protocols. However, it is important to note that there are indeed RF-shim settings, indicated in red in Fig. 5, which result in the actual SAR being higher than that used by the scanner. In most cases, these RF-shim values are quite distant (e.g. a difference in power distribution of up to a factor-of-five) from ones that would be calculated by the scanner either for minimizing B_1_^+^ inhomogeneity or SAR, and thus are very unlikely to be chosen. Nevertheless, as shown by the case of Donna, the difference between an overestimation of 10 % and an underestimation of 10 % can also be relatively small.

One limitation of the current study is the relatively small number of body models studied in the simulations, although we did try to cover as large a range of BMI as is possible using available body models. Experimental data (results not shown) confirmed the work of Krishnamurthy et al. (5) that the relative amplitude and phase settings between the two channels are essentially independent of body habitus. The simulations in Fig. 5 show that also the SAR ratio is quite similar for many different types of body size and composition, although for the highest BMI case there were some noticeable differences. The results presented are essentially independent of whether a standard-bore or wide-bore magnet is used: throughout the manuscript simulations have been presented for the standard-bore system, but simulations performed for the wide-bore system show essentially identical behavior (data not shown). Another experimental degree of variability is the potential incorrect positioning of the high permittivity pads on the subjects’ body. The high permittivity pads are visible with a very low intensity on the localizer scans and so can be repositioned quickly if required. In simulations (data not shown) the SAR over- or underestimation was essentially identical if the offset in pad position was less than 5 cm.

How might the improved performance be translated to a clinical platform while ensuring patient safety? The most pragmatic and simple solution would be to run the system in quadrature mode when pads are being used, since from Tables 1 and 2 one can see that local SAR is consistently reduced when using the pads in quadrature mode, while *B*_1_^+^ homogeneity is typically higher than that of the RF shimmed case without pads. However, this would miss out on the residual reductions in SAR possible by dual channel RF-shimming. A second option would be to introduce an error margin which would reduce the SAR limit when using pads to ~90 % of that when not using pads. This would mean that even if the RF shimming algorithm malfunctioned and produced values corresponding to a “red area” in Fig. 5, then the chances of underestimating SAR are very low. The ultimate method to take full advantage of the high permittivity pads is, of course, to generate a second SAR model used by the scanner in which a fixed size and position of pads have been included.

## Conclusions

The combination of active (dual transmit) and passive (high permittivity pads) RF shimming shows great promise for increasing image quality for cardiac imaging at 3T. The current simulation study shows that transmit performance improves when high permittivity pads are used and RF-shim settings are optimized for producing either the highest degree of *B*_1_^+^ homogeneity or the lowest SAR. Both of these factors enable higher imaging quality to be achieved for cardiac MR at 3T.

## Abbreviations

SAR: specific absorption rate
RF: Radiofrequency
SSFP: Steady state free precession
TR: Repetition time

## References

[1] Oshinski JN, Delfino JG, Sharma P, Gharib AM, Pettigrew RI (2010). Cardiovascular magnetic resonance at 3.0 T: Current state of the art. J Cardiovasc Magn Reson.

[2] Cheng ASH, Pegg TJ, Karamitsos TD, Searle N, Jerosch-Herold M, Choudhury RP, Banning AP, Neubauer S, Robson MD, Selvanayagam JB (2007). Cardiovascular magnetic resonance perfusion imaging at 3-tesla for the detection of coronary artery disease: a comparison with 1.5-tesla. J Am Coll Cardiol.

[3] Thomas D, Strach K, Meyer C, Naehle CP, Schaare S, Wasmann S, Schild HH, Sommer T (2008). Combined myocardial stress perfusion imaging and myocardial stress tagging for detection of coronary artery disease at 3 Tesla. J Cardiovasc Magn Reson.

[4] Gharib AM, Abd-Elmoniem KZ, Herzka DA, Ho VB, Locklin J, Tzatha E, Stuber M, Pettigrew RI (2011). Optimization of coronary whole-heart MRA free-breathing technique at 3 Tesla. Magn Reson Imaging.

[5] Sung K, Nayak KS (2008). Measurement and Characterization of RF Nonuniformity Over the Heart at 3 T Using Body Coil Transmission. J Magn Reson Imaging.

[6] Greenman RL, Shirosky JE, Mulkern RV, Rofsky NM (2003). Double Inversion Black-Blood Fast Spin-Echo Imaging of the Human Heart: A Comparison Between 1.5 T and 3.0 T. J Magn Reson Imaging.

[7] Barkhausen J, Ruehm SG, Goyen M, Buck T, Laub G, Debatin JF (2001). MR evaluation of ventricular function: true fast imaging with steady-state precession versus fast low-angle shot cine MR imaging: feasibility study. Radiology.

[8] Willinek WA, Gieseke J, Kukuk GM, Nelles M, König R, Morakkabati-Spitz N, Träber F, Thomas D, Kuhl CK, Schild HH (2010). Dual-source parallel radiofrequency excitation body MR imaging compared with standard MR imaging at 3.0 T: initial clinical experience. Radiology.

[9] Van den Berg CAT, van den Bergen B, van de Kamer JB, Raaymakers BW, Kroeze H, Bartels LW, Lagendijk JJW (2007). Simultaneous B1+ Homogenization and Specific Absorption Rate Hotspot Suppression Using a Magnetic Resonance Phased Array Transmit Coil. Magn Reson Med.

[10] Katscher U, Börnert P (2006). Parallel RF transmission in MRI. NMR Biomed.

[11] Mueller A, Kouwenhoven M, Naehle CP, Gieseke J, Strach K, Willinek WA, Schild HH, Thomas D (2012). Dual-source radiofrequency transmission with patient-adaptive local radiofrequency shimming for 3.0-T cardiac MR imaging: initial experience. Radiology.

[12] Jia H, Wang C, Wang G, Qu L, Chen W, Chan Q, Zhao B (2013). Impact of 3.0 T Cardiac MR Imaging Using Dual-Source Parallel Radiofrequency Transmission with Patient-Adaptive B1 Shimming. PLoS One.

[13] Krishnamurthy R, Pednekar A, Kouwenhoven M, Cheong B, Muthupillai R (2013). Evaluation of a Subject specific dual-transmit approach for improving B1 field homogeneity in cardiovascular magnetic resonance at 3 T. J Cardiovasc Magn Reson.

[14] Strach K, Clauberg R, Müller A, Wonneberger U, Naehle CP, Kouwenhoven M, Gieseke J, Schild HH, Thomas D (2013). Feasibility of high-dose dobutamine stress SSFP Cine MRI at 3 Tesla with patient adaptive local RF Shimming using dual-source RF transmission: initial results. RöFo.

[15] Haines K, Smith NB, Webb AG (2010). New high dielectric constant materials for tailoring the B1+ distribution at high magnetic fields. J Magn Reson.

[16] Brink WM, Webb AG (2014). High permittivity pads reduce specific absorption rate, improve B1 homogeneity, and increase contrast-to-noise ratio for functional cardiac MRI at 3 T. Magn Reson Med.

[17] Christ A, Kainz W, Hahn EG, Honegger K, Zefferer M, Neufeld E, Rascher W, Janka R, Bautz W, Chen J, Kiefer B, Schmitt P, Hollenbach H-P, Shen J, Oberle M, Szczerba D, Kam A, Guag JW, Kuster N (2010). The Virtual Family—development of surface-based anatomical models of two adults and two children for dosimetric simulations. Phys Med Biol.

[18] Petoussi-Henss N, Zanki M, Fill U, Regulla D (2002). The GSF family of voxel phantoms. Phys Med Biol.

[19] Graesslin I, Homann H, Biederer S, Börnert P, Nehrke K, Vernickel P, Mens G, Harvey P, Katscher U (2012). A Specific Absorption Rate Prediction Concept for Parallel Transmission MR. Magn Reson Med.

[20] Kuehne A, Seifert F, Ittermann B. GPU-Accelerated SAR Computation with Arbitrary Averaging Shapes. In *Proceedings of the 20th Annual Meeting of ISMRM, Melbourne, Australia*; 2012:2735

[21] El-Sharkawy A-MM, Qian D, Bottomley PA, Edelstein WA (2012). A multichannel, real-time MRI RF power monitor for independent SAR determination. Med Phys.

